# Synaptic Vesicle Docking: Sphingosine Regulates Syntaxin1 Interaction with Munc18

**DOI:** 10.1371/journal.pone.0005310

**Published:** 2009-04-23

**Authors:** Paola G. Camoletto, Hugo Vara, Laura Morando, Emma Connell, Fabio P. Marletto, Maurizio Giustetto, Marco Sassoè-Pognetto, Paul P. Van Veldhoven, Maria Dolores Ledesma

**Affiliations:** 1 Department of Molecular and Developmental Genetics, VIB, Leuven, Belgium; 2 Center for Human Genetics, Katholieke Universiteit Leuven, Leuven, Belgium; 3 Department of Molecular Neurobiology, Centro de Biología Molecular Severo Ochoa, CSIC-UAM, Madrid, Spain; 4 Department of Anatomy, Pharmacology and Forensic Medicine and National Institute of Neuroscience-Italy, Turin, Italy; 5 Medical Research Council Laboratory of Molecular Biology, Cambridge, United Kingdom; 6 Department of Molecular Cell Biology, LIPIT, Katholieke Universiteit Leuven, Leuven, Belgium; National Institutes of Health, United States of America

## Abstract

Consensus exists that lipids must play key functions in synaptic activity but precise mechanistic information is limited. Acid sphingomyelinase knockout mice (ASMko) are a suitable model to address the role of sphingolipids in synaptic regulation as they recapitulate a mental retardation syndrome, Niemann Pick disease type A (NPA), and their neurons have altered levels of sphingomyelin (SM) and its derivatives. Electrophysiological recordings showed that ASMko hippocampi have increased paired-pulse facilitation and post-tetanic potentiation. Consistently, electron microscopy revealed reduced number of docked vesicles. Biochemical analysis of ASMko synaptic membranes unveiled higher amounts of SM and sphingosine (Se) and enhanced interaction of the docking molecules Munc18 and syntaxin1. *In vitro* reconstitution assays demonstrated that Se changes syntaxin1 conformation enhancing its interaction with Munc18. Moreover, Se reduces vesicle docking in primary neurons and increases paired-pulse facilitation when added to wt hippocampal slices. These data provide with a novel mechanism for synaptic vesicle control by sphingolipids and could explain cognitive deficits of NPA patients.

## Introduction

Increasing evidence suggests a key role for lipids in the establishment and functionality of synapses. Thus, glial-derived cholesterol was identified as an essential factor promoting synapse formation [1] whereas pharmacological reduction of cholesterol and sphingolipids levels leads to synapse loss [2]. Phosphoinositides [3] and cholesterol [4] regulate the synaptic vesicle cycle. Moreover, arachidonic and phosphatidic acids stimulate vesicle fusion by interacting with the SNARE exocytic complex [5]–[7]. However, much still remains to be learned about the mechanisms by which lipids influence synaptic function and about the enzymatic activities regulating their action. This knowledge is essential to understand not only the molecular mechanisms of cognition but also the defects underlying the cognitive impairment that accompany most lipidosis. Among them, NPA results from loss of function mutations in the acid sphingomyelinase (ASM) gene leading to severe mental retardation [8]. ASM is the enzyme responsible for the conversion of SM into ceramide in the lysosomes [9]. Its absence causes the accumulation of SM in these organelles, which is a hallmark in NPA patients. SM-loaded lysosomes also characterize the cells of ASMko mice [10], [11]. These mice recapitulate the human disease symptoms showing axonal dystrophy [12] and neurodegeneration, particularly dramatic in cerebellar Purkinje cells [13]. Furthermore, accumulation of SM and its derivatives also occurs at the plasma membrane of hippocampal neurons [14]. This moved us to consider these mice a suitable model to investigate the involvement of sphingolipids in synaptic function.

## Results

### The absence of ASM causes a drastic increase of SM in synaptic membranes

To determine whether lack of ASM activity affects synaptic membrane lipid composition, mass analysis of lipids was performed on synaptosomes of age-matched (7months) wild type (wt) and ASMko mice. The synaptosome isolation procedure was refined so that no traces of myelin and lysosomes, which are loaded with SM in ASMko conditions, could interfere with our measurements (see methods and Supplementary Figure S1). This study revealed a 3-fold increase in SM levels in ASMko synaptosomes compared to wt (176±40 and 59±17 nmol/mg protein, respectively, mean±SD, n = 3). In contrast, the amounts of other lipids such as cholesterol (362±66 and 270±53 nmol/mg protein), ceramide (3.2±0.1 and 2.7±1 nmol/mg protein), phosphatidylcholine (306±107 and 266±79 nmol/mg protein), phosphatidylserine (64±28 and 38±6 nmol/mg protein) and phosphatidylethanolamine (247±74 and 200±29 nmol/mg protein), were not significantly altered. These results evidence that ASM deficiency alters the synaptic membrane lipid composition by drastically increasing SM levels.

### ASM deficiency leads to the alteration of short-term synaptic plasticity events

**Figure 1:**
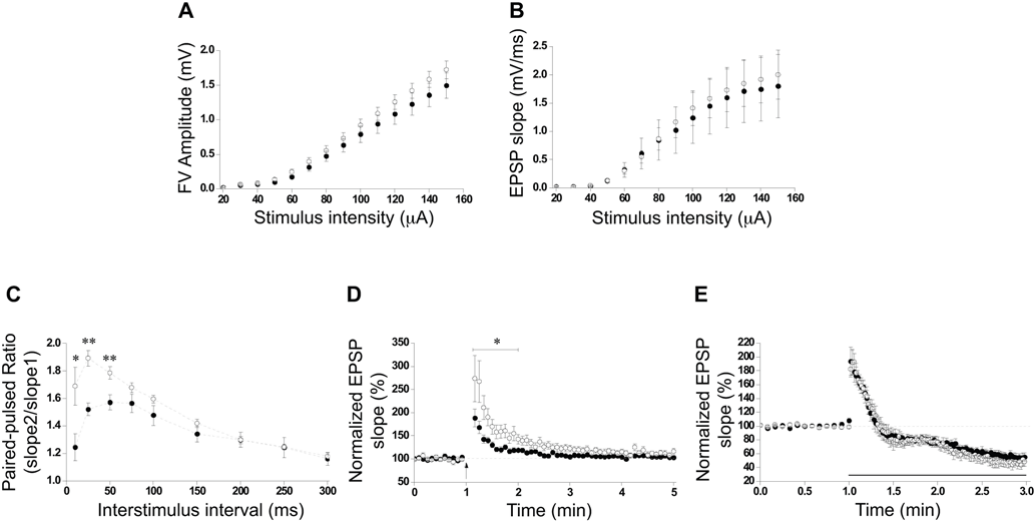
Basal synaptic transmission and plasticity in wt and ASMko hippocampal slices. (A,B) Input-output plots of stimulus versus FV amplitude (A) and stimulus versus fEPSP slope (B) in wt and ASMko slices stimulated at intensities ranging from 10 to 150 µA. (C) PPF recording expressed as mean paired-pulse ratios of fEPSPs at nine interstimulus intervals in wt and ASMko slices. (D) PTP plot in wt and ASMko slices. Arrows indicate tetanus application (100 Hz for one second). (E) Depression induced by high-frequency stimulation in wt and ASMko slices. The solid line indicates the period of stimulation at 10 Hz. For clarity, only one every ten measurements is plotted during 10-Hz stimulation. For all graphs n = 10 slices per condition. Solid circles: wt slices; open circles: ASMko slices. Statistical significance: *p<0.05; ** p<0.01.

**Figure 2:**
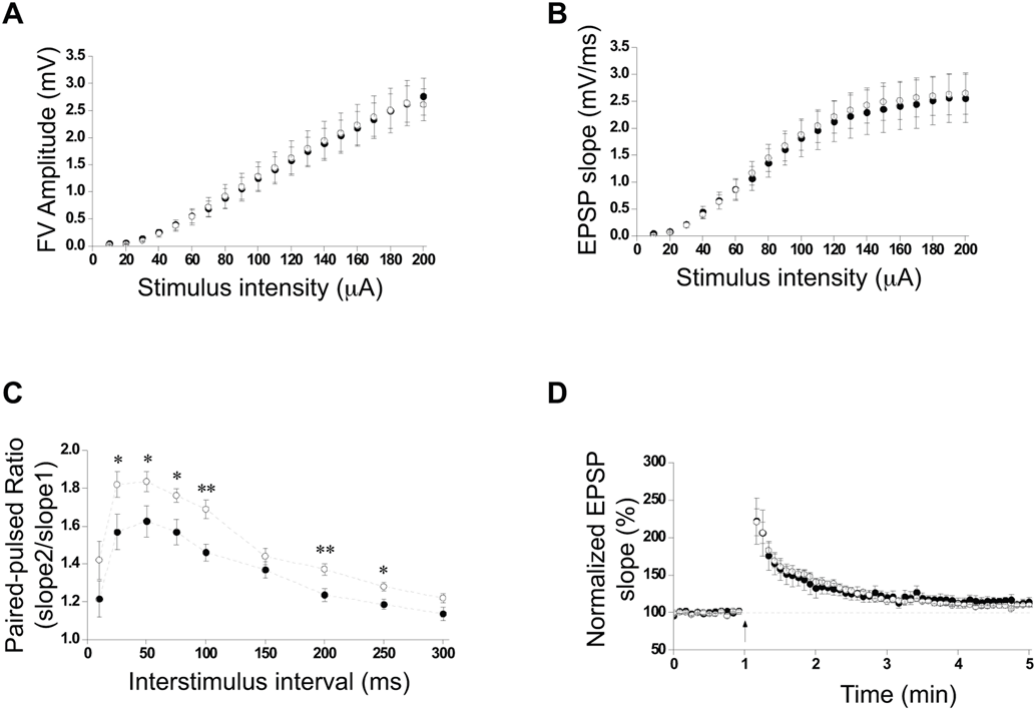
Basal synaptic transmission and plasticity in wt hippocampal slices treated or not with SM. (A,B) Input-output plots of stimulus versus FV amplitude (A) and stimulus versus fEPSP slope (B) in wt vehicle-treated and wt SM-treated slices stimulated at intensities ranging from 10 to 200 µA. (C) PPF recording expressed as mean paired-pulse ratios of fEPSPs at nine interstimulus intervals in wt vehicle-treated and wt SM-treated slices. (D) PTP plot in wt vehicle-treated and wt SM-treated slices. Arrows indicate tetanus application (100 Hz for one second). For all graphs n = 10 slices per condition. Solid circles: wt vehicle-treated slices; open circles: wt SM-treated slices. Statistical significance: *p<0.05; ** p<0.01.

Alterations in the lipid composition of the synapses could affect synaptic transmission. To test if this is the case when ASM activity is lacking, synaptic transmission and short-term synaptic plasticity of Schaffer collateral (Sc)-CA1 synapses were next investigated in hippocampal slices from 7-month-old wt and ASMko mice. Basal synaptic transmission measured by input-output analysis was normal in ASMko mutants (Figure 1A,B). In contrast, short-term synaptic plasticity in ASMko slices showed enhanced paired-pulse facilitation (PPF) (Figure 1C) and post-tetanic potentiation (PTP) (Figure 1D), while synaptic depression remained unaltered (Figure 1E). To determine whether the increased levels of SM in ASMko conditions could explain the electrophysiological alterations, we assessed the effects of the addition of exogenous SM to slices of wt mice. Input-output analysis of SM-treated slices showed no differences with respect to controls (Figure 2A,B). However, PPF ratio was increased mimicking the results obtained in ASMko slices (Figure 2C). We could not detect abnormalities in PTP under our experimental conditions (Figure 2D). This could be due to the high dependence of PTP on events occurring inside the presynaptic terminal (i.e. mitochondrial calcium buffering), which would not be affected if the exogenously added SM only reaches the plasma membrane. In any case, these results indicate that the accumulation of SM is responsible, at least in part, for the alterations in presynaptic function observed in ASMko mice. This is also supported by the fact that the lipid increase precedes these alterations. Thus, we registered normal PTP and PPF in hippocampal slices from ASMko mice of 1 month of age (Supplementary Figure S2A), when symptoms of the disease are not yet evident but SM increase is already detectable. However, this increase is only 0.6-fold of that occurring at 7 months (126±13 and 192±14 nmol/µmol phospholipids in 1- and 7- months-old ASMko, respectively). This suggests that a certain threshold in SM accumulation must be achieved for the aberrant phenotypes to occur during the progression of the disease.

### Lack of ASM results in smaller synapses and reduced number of docked vesicles

**Figure 3:**
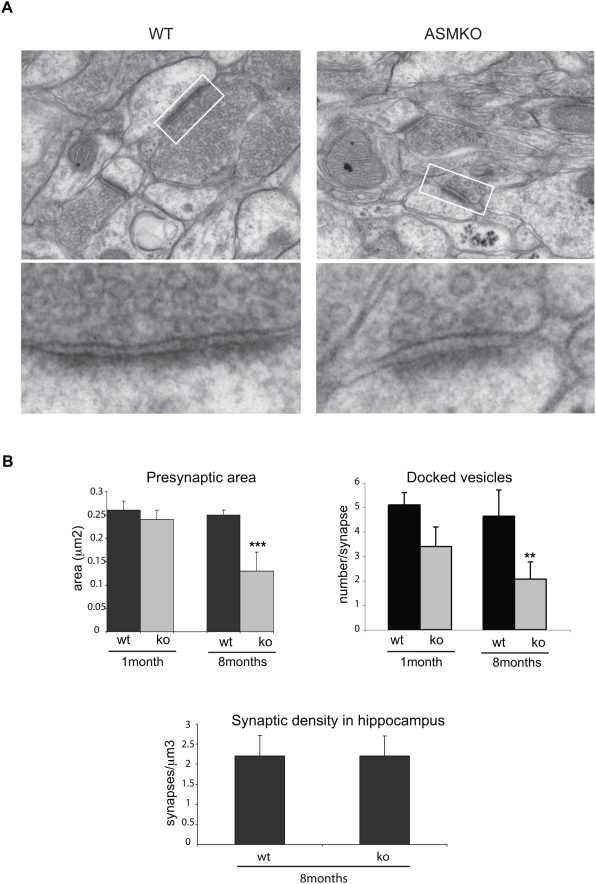
Smaller size of the presynaptic compartments and reduced number of docked vesicles in ASMko mice hippocampus compared to wt. (A) Representative electron micrographs showing axospinous synapses in the CA1 stratum radiatum of wt and ASMko mice. Insets show at higher magnification docking synaptic vesicles at the presynaptic active zone. (B) Graphs show mean±SD (n = 3 mice per genotype and age) of the following parameters: size of presynaptic boutons, number of docked vesicles, density of axospinous synapses, **p<0.01; *** p<0.001.

The above reported changes in short-term presynaptic plasticity in ASMko hippocampus are consistent with a decrease probability of neurotransmitter release [15]. Since the number of docked vesicles is a morphological correlate of neurotransmitter release probability at hippocampal synapses [16], we next analyzed by electron microscopy presynaptic terminals in stratum radiatum of the CA1 region (Figure 3A). No significant differences in ultrastructure were found in 1-month-old mice. However, a reduction in the size of the presynaptic area in ASMko mice was noticeable at 2 and 5 months being most significant in symptomatic 8-month-old mice (Figure 3B). Additionally, the total number of synaptic vesicles was diminished in ASMko mice (ASMko 51.4±4.2, wt 71.7±6.6) although their density was not altered with respect to wt synapses (ASMko 295.3±16.95 vesicles/µm^2^, wt 304.7±44.2 vesicles/µm^2^), given the smaller size of ASMko presynaptic boutons. Notably, while the number of docked vesicles was not significantly reduced at 1 month of age the reduction was evident in 8 month old ASMko mice (Figure 3B) (number of docked vesicles per synapse: ASMko 2.07±1.07, wt 4.64±1.70); number of docked vesicles per µm of active zone: ASMko 8.84±2.76, wt 16.98±3.2). Despite these changes, the general appearance of the neuropil was normal, the only detectable sign of degeneration being the presence of lipid inclusions in the cell body of pyramidal neurons. These inclusions were already significant at 1 month of age filling the cytoplasm at 8 months (Supplementary Figure S3). Stereological methods revealed a similar density of axospinous synapses in the stratum radiatum of both genotypes at 8 months of age (Figure 3B). Taken together, these data indicate that although synapses are retained in the hippocampus of ASMko mice even at advanced stages of the disease, there is a marked decrease in the dimension of presynaptic specializations and in the pool of docked vesicles.

### ASM deficiency alters the interaction of the docking molecules Munc18 and Syntaxin1

**Figure 4:**
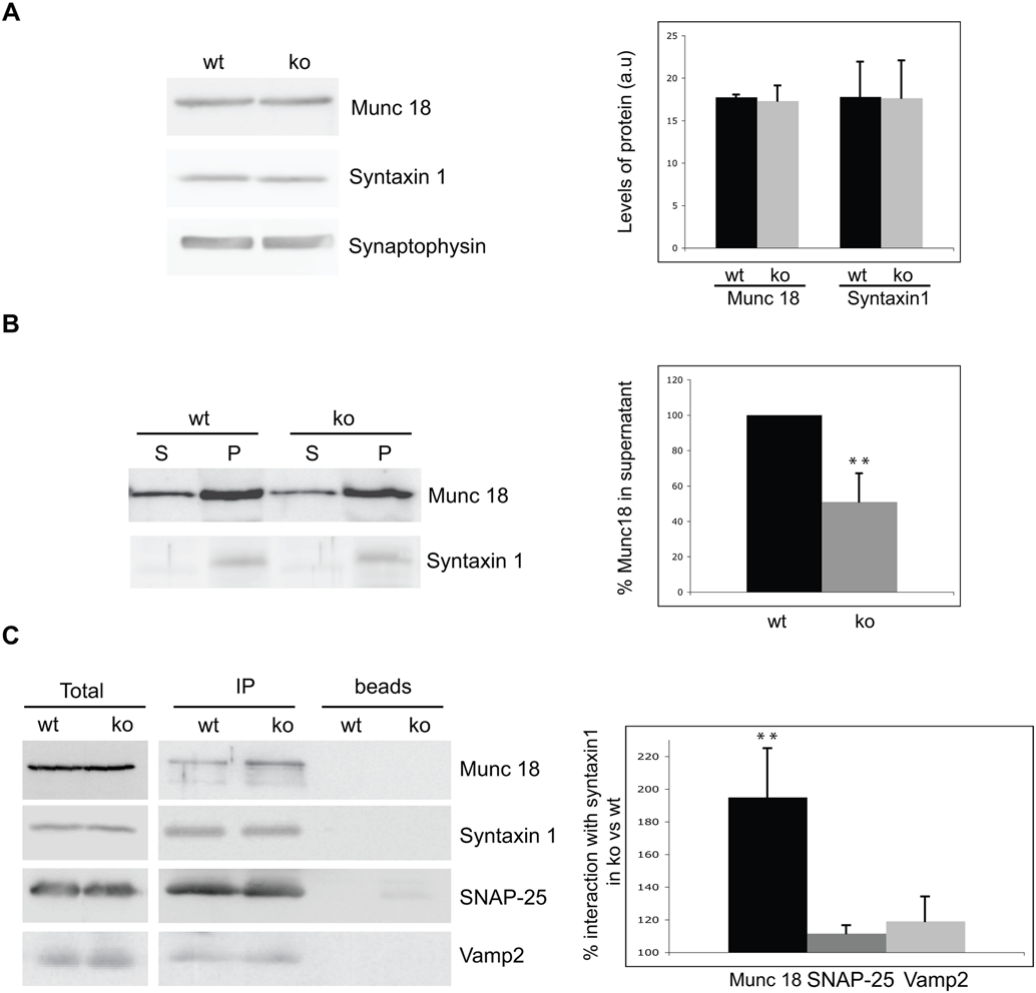
Altered interaction of Munc18 and Syntaxin1 in ASMko synaptosomes. (A) Total levels of Munc18 and syntaxin1 analyzed by Western blot in synaptosomal samples from 7 month-old wt and ASMko mice containing the same amount of protein. Levels of synaptophysin were used as loading control. Graph shows mean±SD (n = 3 brains per genotype) in arbitrary units. (B) Amounts of Munc18 and syntaxin1 in the supernatant (S) and pellets (P) after 100000 g centrifugation of wt and ASMKO synaptosomal lysates containing equal amount of protein. Graph shows mean±SD (n = 3) as Munc18 ratio between supernatant and pellet. (C) Amount of Munc18, SNAP25, Vamp2 and syntaxin1 immunoprecipitated from equal amounts of ASMko and wt synaptosomes (shown in the total extracts) incubated (IP) or not (beads) with the antibody against syntaxin1. Graph shows mean±SD (n = 3) as percentage of interaction of Munc 18, SNAP25 or Vamp2 with syntaxin1 in ASMko versus wt samples. **p<0.01.

Syntaxin 1 and Munc 18 have been postulated as a docking platform for secretory vesicles [17]. Accordingly, Munc18 protein levels and its binding to the membrane through its interaction with syntaxin1 influence the docking process [18], [19], [20], [21], [22]. To gain insight into the molecular mechanisms that could impair synaptic vesicle docking in ASMko mutants we analyzed Munc18 and syntaxin1 expression levels, membrane attachment and interaction in synaptosomes from 7-month-old wt and ASMko mice. While total levels were similar, the pool of Munc18 associated to membranes was significantly increased in ASMko synaptosomes (Figure 4A,B). Consistently, immunoprecipitation assays revealed that the interaction of Munc18 with syntaxin1 was 1.9-fold higher in ASMko conditions (Figure 4C). Importantly, Syntaxin1-Munc18 interaction was not altered in synaptosomes from 1-month-old ASMko mice (Supplementary Figure S2B), in which vesicle docking defects are not yet significant. Finally, to address whether the interaction of syntaxin1 with the other core components of the presynaptic exocytic complex was affected or not immunoprecipitates were analyzed for the presence of SNAP25 and Vamp2/synaptobrevin. No differences were observed for SNAP25 and although the amount of Vamp2 showed a tendency to increase this was not statistically significant in ASMko synaptosomes compared with wt (Figure 4C). These findings show that lack of ASM results in anomalous membrane binding and interaction with syntaxin1 of Munc18 at advanced stages of the disease when the docking alterations are evident.

### The SM derivative Se induces a drastic conformational change in Syntaxin 1

**Figure 5:**
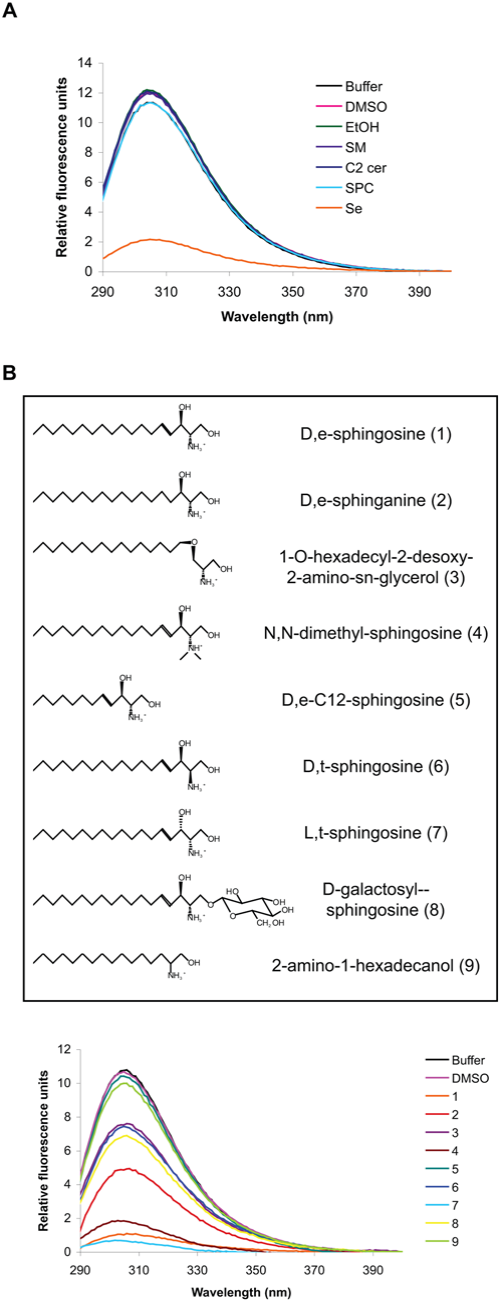
Se alters the conformation of syntaxin1. (A) Fluorescence spectra of syntaxin1 at excitation of 280 nm in the presence of 100 µM SM, N-acetyl-Se (C2-cer), Se, SPC or the different solvents used to solubilized the lipids (buffer, DMSO and ethanol). (B) Changes in the fluorescence spectra of syntaxin1 upon addition of the different Se analogues (100 µM). Upper panel shows the structures of the analogues (codes in parenthesis).

Our next aim was to understand to which extent the excess of sphingolipids in the ASMko synaptic membranes was responsible for the altered syntaxin1/Munc18 interaction. Munc18 can bind to different conformational states of syntaxin1 with distinct affinity [22], [23]. Hence, we tested whether the excess in SM found in ASMko synaptosomes induces a change in syntaxin1 conformation, which could lead to the increased binding of Munc18. To this aim we measured the intrinsic fluorescence of recombinant syntaxin1 [24] (Supplementary Figure S4) in control conditions and upon addition of SM. No changes were observed (Figure 5A). Given that increased levels of different SM derivatives, namely ceramide, sphingosylphosphorylcholine (SPC) and Se, had been found at the plasma membrane of ASMko neurons [14], we next tested whether these lipids could alter syntaxin1 conformation. Only Se had a strong effect in the intrinsic fluorescence of syntaxin1 (Figure 5A). To define the precise mechanism of action of Se, we analyzed syntaxin1 conformational changes upon addition of different Se analogues (Figure 5B). These experiments revealed that the substitution of the free primary hydroxygroup of Se was allowed with a neutral group (D-galactosyl sphingosine is active) but not with a charged group (SPC is inactive). The double bond of Se was not necessary to induce a conformational change as D,e-sphinganine was still able to alter the intrinsic fluorescence of syntaxin 1(Figure 5B). However, 2-amino-1-hexadecanol, resembling sphinganine but lacking the secondary hydroxygroup, was ineffective. On the other hand, the configuration of this hydroxygroup seemed less critical (both D,erythro- and L,threo-sphingosine being active). The length of the hydrophobic tail was important as C12-sphingosine failed to change syntaxin1 conformation compared to Se (Figure 5B). Finally, the free aminogroup (N-acetylation resulted in an inactive compound) and its configuration (D,threo-sphingosine has less effect), did control the interaction with syntaxin1 (Figure 5B). Interestingly, alkylation of the aminogroup was allowed (N,N-dimethyl-sphingosine is still active) suggesting that it is not the aminogroup itself but the positive charge that is important to induce conformational changes (Figure 5B). The differential effects obtained with the diverse analogues used in the same experimental conditions (i.e. concentration, solvent, temperature, time of incubation) strongly support that the alterations in fluorescence are not due to protein denaturation or fluorescence quenching but to specific conformational changes. Altogether, these data point to Se as a physiological regulator of syntaxin1 conformation and shed light on the structural requirements for their interaction.

### High Se levels are present in ASMko synaptosomes, alter Munc18-syntaxin1 interaction, reduce the ready releasable pool of vesicles and increase PPF

**Figure 6:**
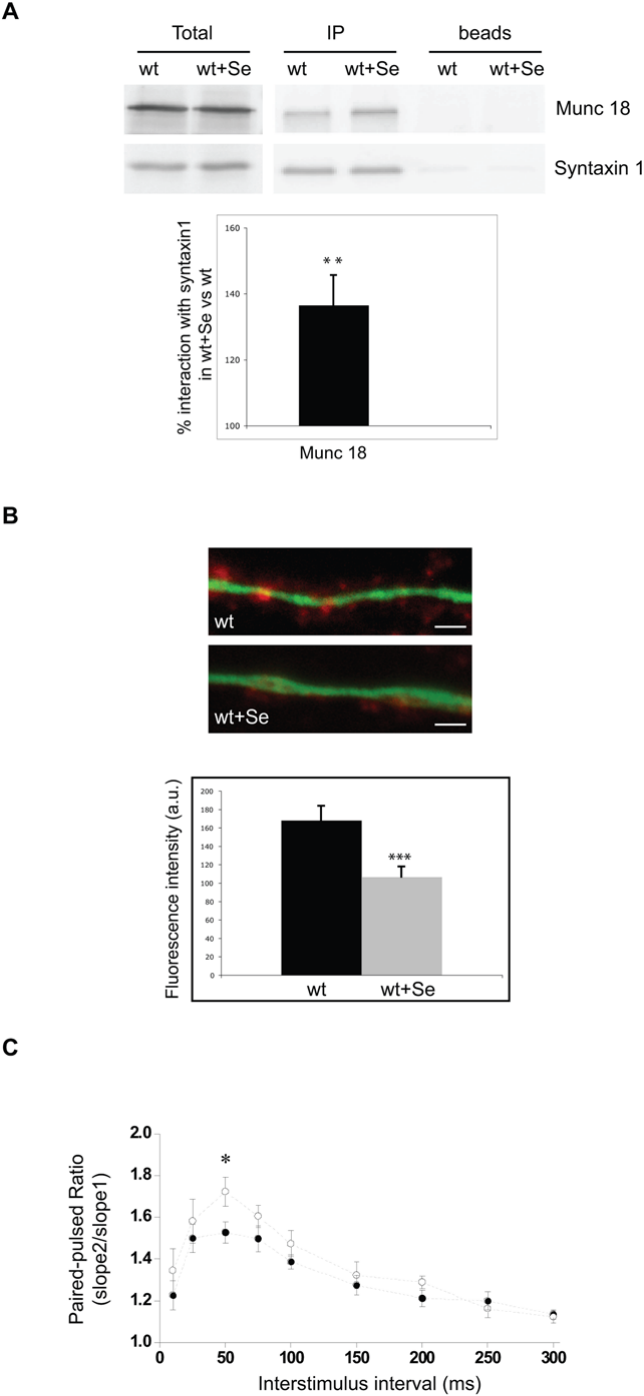
Se alters syntaxin1 interaction with Munc18, the ready releasable pool of vesicles and PPF. (A) Amount of Munc18 and syntaxin1 immunoprecipitated from equal amounts of wt synaptosomes (shown in the total extracts) treated or not with Se, using an antibody against syntaxin1. Graph shows mean±SD (n = 3) as percentage of interaction of Munc 18 with syntaxin1 in wt+Se versus wt samples, **p<0.01. (B) Examples of presynaptic terminals, labeled with FM4–64 (red) by using 500 mM sucrose, on dendrites stained with an antibody against MAP2 (green) in wt cultured hippocampal neurons treated or not with 50 µM Se. Bar = 5 µm. Graph shows mean±SD of the fluorescence associated to 500 synapses on 15 neurons per condition expressed as arbitrary units, ***p<0.001. (C) PPF recording expressed as mean paired-pulse ratios of fEPSPs at nine interstimulus intervals in wt vehicle-treated and wt Se-treated slices (n = 10 slices per condition). Solid circles: wt vehicle-treated slices; open circles: wt Se-treated slices. Statistical significance: *p<0.05.

To address whether increased Se levels could account for the anomalies in synaptic vesicle docking and presynaptic plasticity in ASMko mice we first tested whether the amount of this lipid was increased at the synaptic membrane, similarly to total membranes [14]. Indeed, ASMko synaptosomes from 7-month-old mice contained 4–5 fold more Se than wt (266.8±102.8 and 59.6±15.1 pmol/mg protein, respectively, n = 3). Moreover, treatment of wt synaptosomes with Se, which raised Se levels without affecting other lipids (Supplementary Figure S5), enhanced 1.36-fold Munc18-syntaxin1 interaction (Figure 6A). To directly prove that high Se levels impair vesicle docking we labeled the ready releasable pool (RRP) of vesicles in cultured hippocampal neurons by using 500 mM sucrose solution containing the fluorescent dye FM4–64 [18]. This experiment revealed a significant 36% fluorescence reduction in wt neurons treated with Se compared to non treated neurons (Figure 6B). Finally, to demonstrate that Se accumulation results in the alteration of presynaptic plasticity we measured PPF in hippocampal slices of wt mice treated or not with Se. PPF increased upon Se addition similarly to the behavior observed in slices from ASMko mice (Figure 6C). These observations confirm Se as a modulator of Munc18-syntaxin1 interaction, vesicle docking and presynaptic plasticity. They also indicate that increased levels of this lipid contribute to the docking and electrophysiological defects observed in neurons from 7–8-month-old ASMko mice. Consistently, Se increase precedes these alterations being already detectable in 1-month-old ASMko mice, in which such defects are not significant (Supplementary Figure S2A,B). However, the increase is only 0.6-fold of that occurring at 7 months (194±21 and 286±30 pmol/µmol phospholipids in 1-month-old and 7-month-old-mice, respectively), suggesting that, as with SM, a certain threshold in the levels of Se must be achieved for the aberrant phenotypes to appear.

## Discussion

Our data, obtained using a variety of experimental approaches including electrophysiological, electron microscopy, biochemical and biophysical analysis, contribute to define the molecular mechanisms involved in synaptic vesicle docking. Although the involvement of complexes of SNARE and Munc18 in such process is established, the need to identify factors involved in the control of the conformation or binding capabilities of these proteins has been acknowledged [17]. We here show that SM, Se and their regulatory enzyme ASM, are among these factors. Thus, when ASM is lacking, SM accumulates leading to high levels of its derivative Se. We demonstrate that Se increase affects presynaptic plasticity events, impairs vesicle docking in primary neurons and alters the interaction of the docking molecules Munc18-syntaxin1 by changing syntaxin1 conformation. We also offer insight on how this occurs along the progression of NPA disease. Hence, Se increase precedes these defects that appear when the levels of this lipid reach a certain threshold. Furthermore, we provide detail on the structural requirements in the Se molecule necessary to change syntaxin1 conformation, which could open perspectives for future drug design. Such an effect had been only shown for arachidonic acid [24]. However, the latter favors the open, fusion competent, conformation of syntaxin1 without affecting Munc18-syntaxin1 interaction [24]. Because we observe that Se enhances their contact without changing the levels of these proteins, we propose that this lipid promotes the closed conformation of syntaxin1, which has the highest affinity for Munc18 [22]. Hence, the balance of arachidonic acid and Se levels at the synaptic membrane could be a key factor for docking and fusion by differentially modulating syntaxin1 conformation.

Our results showing an increased interaction of Munc18-syntaxin1 yet reduced number of docked vesicles, are in agreement with the proposed role of Munc18 in controlling the size of the ready releasable pool of vesicles [18]. However, they are in apparent contradiction to the view that the formation of the Munc18-syntaxin1 complex correlates directly with the docking process [17], [20], [21], [22]. Considering that we move in a disease scenario, it might still be that altered Se levels all over the synaptic ASMko membrane sequester Munc18, through enhanced syntaxin1 interaction, to membrane areas different from the active zone and incompetent for docking.

Finally, our work provides with the functional consequences that the lack of ASM have in short-term presynaptic plasticity events. Anatomical analysis offered insight on the events underlying the functional observations. Thus, that the density of the reserve pool vesicle store is not affected in ASMko conditions could explain why synaptic depression is not altered. The finding that the basal response is also not affected in ASMko synapses, despite the reduction in the ready releasable pool of vesicles and the increase in PPF, is surprising. However, several facts could explain this observation. First, the density of neurotransmitter receptors (NT Rcs) has been proposed as a key element for the basal synaptic response [25]. We have shown that ASMko synapse is smaller, however biochemical analysis of wt and ASMko synaposomal preparations revealed similar levels of NT Rcs (unpublished observations). Hence, it is possible that in such conditions a higher density of NT Rcs at the postsynaptic compartment compensate for the reduced presynaptic release, rendering the basal response as efficient as in the wt situation. Moreover, the absence of neurodegeneration and the presence of a normal density of axospinous synapses would also contribute to the normal basal synaptic transmission in the hippocampus. Likewise, although the membrane attachment of Munc18 is altered, it has been shown that reduction or overexpression of Munc 18 do not affect the basal synaptic strength [18]. In contrast, increased PPF and PTP are consistent with the scarcity of docked vesicles, which could imply a reduction in basal probability of neurotransmitter release, favouring the enhancement of synaptic strength after repeated stimulation. Because abnormalities in such events have been linked with learning impairment [26] our results could explain, at least in part, the severe mental retardation that characterizes NPA patients in which ASM is missing.

## Materials and Methods

### Materials

Antibodies against the following molecules were used: Lamp-2 (CD 107b, BD Transduction Laboratories); Synaptophysin (Clone Sy-38, Boehringer Mannheim); Myelin Basic Protein (D-18, sc 13912 Santa Cruz); Munc 18 (116002 polyclonal, SySy); Syntaxin1 (110101 monoclonal, SySy); SNAP25 (111002 polyclonal SySy); Vamp2 (104211 monoclonal SySy). The FM4–46 dye was obtained from Molecular probes. Sphingenine stereoisomers, sphinganine and analogues were obtained from Sigma, Bachem or Toronto Research Chemicals, or synthesized [27].

### Mice

A breeding colony of ASM heterozygous C57BL/6 mice (10), kindly donated by Dr. E. Schuchman (Mount Sinai School of Medicine, New York), was established. The experiments were performed by comparing littermates of age matched wt or ASMko mice. All procedures involving the use of animals were performed according to guidelines specified for the animal protection and welfare by the Belgian and Spanish Ministeries of Health and Agriculture, respectively.

### Neuronal cultures

Cultures of hippocampal neurons were prepared from brains of 16-day-old mice embryos [28]. For our experiments hippocampal neurons were kept in culture for more than 12 days when they have established functional synapses.

### Isolation of synaptosomes

Synaptosomes from ASMko and wt mice brains were obtained using Percoll gradients [29]. Two low speed centrifugations (9000×g, 10 min) were included as initial steps after brain homogenization to eliminate lysosomal contamination.

### Mass analysis of lipids

Lipid extracts were prepared as described [14] and analyzed for phospholipids (organic phosphate) [30] and for ceramide by means of recombinant ceramide kinase [31] or subjected to TLC (0.25 mm Silica gel 60, Merck; solvent hexane/diethylether/acetic acid 70/30/1, v/v) followed by elution and enzymatic quantification of cholesterol [32]. Main phospholipids, also separated by TLC (solvent chloroform/methanol/formic acid 65/25/10, v/v), were visualized by iodine staining followed by ashing and phosphate analysis [30]. Recovery of standards after chromatography is estimated at 80–85%. To analyze sphingosine, acidic methanolic extracts were fortified with C_17_-sphingenine (Toronto Research Chemicals), diluted with water and applied to a hydrophobic SPE cartridge (60 mg HLB-Oasis, Waters). Compounds, eluted with methanol, were derivatized with 6-aminoquinolyl-N-hydroxysuccinimidyl carbamate and subjected to normal phase SPE (100 mg NH2-BondElut, Varian) to isolate the derivatized sphingoid bases. After two selective hydrolysis steps, samples were separated by reversed phase HPLC (Symmetry C18-column 4.6×150; 5 µm; 100Å; Waters) with an increasing gradient of buffered methanol/acetonitrile coupled to fluorimetric analysis (Van Veldhoven P.P., to be published).

### Electron microscopy analysis

Mice were anesthetized with an intraperitoneal injection of ketamine-xylazine 1∶1 (0.1 ml/kg) and perfused through the left ventricle with a mixture of paraformaldehyde (4%) and glutaraldehyde (2%) in phosphate buffer (PB, 0.1 M, pH 7.4). The brains were removed from the skull and postfixed in the same solution for 4 h. The hippocampal formation was dissected free and its dorsal half was cut into transverse slabs that were postfixed in 1% osmium tetroxide (in 0.1 M cacodylate buffer), dehydrated in ethanol and embedded in Epon-Araldite. The slabs were trimmed in order to obtain serial ultrathin sections of the CA1 region, which were collected on pioloform-coated, single-hole grids, and stained with uranyl acetate and lead citrate. The sections were analysed with a JEM-1010 transmission electron microscope (Jeol, Japan) equipped with a side-mounted CCD camera (Mega View III, Soft Imaging System, Germany). Semithin sections were collected on glass slides, stained with toluidine blue and analysed with a Nikon Eclipse 800 microscope equipped with a CCD camera. Ultrathin sections were collected on pioloform-coated, single-hole grids, and stained with uranyl acetate and lead citrate. Synapse density was estimated using the physical disector method on micrographs of adjacent serial sections taken in the proximal part of stratum radiatum at a magnification of ×20.000. In each mouse (n = 3 mice per group), 40 disectors were examined in two distinct pairs of consecutive sections, making a total volume of 79 µm^3^. A morphometric analysis of pre- and postsynaptic specializations was done on electron micrographs taken at ×75.000 (∼70 synapses per mouse, n = 3 mice per group). The area of axonal boutons and dendritic spines, and the length and thickness of the postsynaptic density were measured with the AnalySIS software (Soft Imaging System). The total number of synaptic vesicles and the number of docked vesicles were counted manually in presynaptic boutons establishing axospinous synapses. Docked vesicles were defined as vesicles that were in physical contact with the presynaptic plasma membrane.

### Hippocampal slice preparation and electrophysiology

Age-matched ASMko and wt mice (1 month or 7 months of age) were sacrificed by cervical dislocation. Brains were extracted and the hippocampi were quickly dissected out in ice-cold oxygenated artificial cerebrospinal fluid (ACSF) containing the following (in mM): 120 NaCl, 2.5 KCl, 2.5 CaCl_2_, 1.2 MgCl_2_, 26.2 NaHCO_3_, 1.0 NaH_2_PO_4_, 11.0 glucose, bubbled with a mixture of 95% O_2_ / 5% O_2_, pH 7.4. Transverse hippocampal slices (400 µM) were cut with a manual tissue chopper. After dissection, slices were maintained in a humidified holding chamber at room temperature for at least one hour before starting electrophysiological recordings. For the experiments in which exogenous SM was added, slices were separated in two groups, one was incubated with SM (0.2 mM) or Se (400 µg/ml) diluted in ethanol (0.8‰ final concentration), while the control group was treated only with vehicle. Both groups were maintained in the holding chamber for at least three hours. Slices were then transferred to an interface recording chamber, where they were continuously perfused with ASCF maintained at 29°C, with a flowing rate of 1.5–2 ml/min. Extracellular field excitatory postsynaptic potentials (fEPSP) were recorded in stratum radiatum of the CA1 hippocampal region with glass electrodes filled with 3 M NaCl coupled to the headstage of an IE-210 electrometer (Warner, USA). A concentric bipolar stainless steel electrode (FHC, USA) was also placed in stratum radiatum, 250–300 µm from the recording electrode, for stimulating the Schaffer collateral afferents (0.1 ms pulse duration). Test stimuli were applied at a frequency of 0.1 Hz for PPF and input-output experiments and 0.2 Hz for PTP and synaptic depression experiments, with stimulus intensities eliciting half maximal fEPSP amplitudes. To ensure stability of the response, test responses were recorded for at least 15 min before the beginning of the experiments. Input/output experiments were performed by stimulating Schaffer afferents at a wide range of intensities, from 10 to 200 µA. Paired-pulse facilitation (PPF) was induced by pairs of stimuli with interstimulus intervals (ISI) ranging from 10 to 300 milliseconds. Post-tetanic potentiation (PTP) was elicited by the application of a tetanic stimulation consisting of a single 100-Hz tetanus for one second. Synaptic depression was examined using a sustained high frequency stimulation (10 Hz) for two minutes. In PTP and synaptic depression experiments, NMDA blocker APV (50 µM) was used to prevent the induction of NMDA-dependent long-term plasticity. Data were collected (10 KHz sampling rate) and analyzed online using Clampex 9.0 software (Axon Instruments, USA). Synaptic activity was measured as the maximal slope of the rising phase of the fEPSP, while presynaptic excitability was calculated as the amplitude of the fiber volley (FV) to its negative peak. Data are presented as means±standard deviation. Statistical analysis was carried out using one-way ANOVA test and an effect was considered significant if p<0.05.

### Munc18-syntaxin1 immunoprecipitation

Synaptosomes from wt, ASMko or wt+Se containing equal amount of protein were brought to a final concentration of 150 mM NaCl, 0.5% sodium deoxycolate and 1% NP40 and precleared for 2 hours at 4°C with protein G sepharose beads. Beads were eliminated by centrifugation and extracts were incubated with syntaxin1 monoclonal antibody for 4 hours at 4°C. Equal amounts of freshly prepared beads were added to the samples and rotated overnight at 4°C. Beads were extensively washed before collecting them for Western blot analysis using anti syntaxin1,Munc 18, SNAP25 and Vamp2 antibodies. Western blot quantification was done with the NIH program in conditions of non-saturated signal. Statistical significance was determined by the student t-test (p<0.05).

### Fluorescence assays

For the intrinsic fluorescence measurements we used recombinant syntaxin1A (aa 1–261) purified as a bacterially-expressed GST-fusion protein with a subsequent GST-removal step (Figure SB). Syntaxin1 fluorescence spectra were analyzed at protein concentration of 0.15 mg/ml and the effects of the addition of SM in 2.5% ethanol, SPC in buffer and C2-ceramide and Se analogues in 2.5% DMSO, at final concentrations of 100 µM, were monitored in a Hellma quartz cuvette. The autofluorescence spectra were collected on a Jobin-Yvon SPEX Fluoromax II fluoremeter with excitation at 280 nm.

### FM4–46 uptake by cultured hippocampal neurons treated or not with Se

Cultured neurons were incubated or not with 50 µM Se for 4 hours. The treatment did not induce cell death as monitored by the TUNEL assay (11±2 and 9±3% of apoptotic neurons in wt and wt+Se cultures, respectively, n = 200 neurons per condition). FM4–46 uptake was conducted as in 18. Briefly, cells were incubated for 3–4 sec with calcium-free Tyrode's containing 16 M FM4–64, 500 mM sucrose, 2.5 mM KCl, 119 mM NaCl, 3 mM MgCl2, 30 mM glucose and 25 mM Hepes, pH 7.4. The 500 mM sucrose-containing solution was replaced by calcium-free Tyrode's containing 16 M FM4–64 for an additional 60 s to ensure labeling of all exocytosed vesicles. Cells were washed for 10 min with calcium-free Tyrode's.

## Supporting Information

Figure S1 Characterization of the Different Fractions of the Protocol Used to Isolate Synaptosomes from ASMKO Mice Brains. Western blots using antibodies against the synaptic marker synaptophysin, the myelin marker Myelin basic protein (MBP) and the lysosomal marker LAMP 2, in samples containing equal amount of protein.
(6.43 MB EPS)

Figure S2 Characterizacion of phenotypes in 1-month-old ASMko mice. A. Hippocampal presynaptic plasticity in 1-month-old mice. (a) PPF expressed as mean paired-pulse ratios of fEPSPs recorded at nine interstimulus intervals in wt and ASMko slices. (b) PTP plot in wt and ASMko slices. Arrows indicate tetanus application (100 Hz for one second). For all graphs n = 10 slices per condition. Solid circles: wt slices; open circles: ASMko slices. B. Syntaxin1- Munc18 interaction in 1-month-old mice. Amount of Munc18 and syntaxin1 immunoprecipitated from equal amounts of ASMko and wt synaptosomes (shown in the total extracts) incubated (IP) or not (beads) with the antibody against syntaxin1. Graph shows mean±SD (n = 3) as percentage of interaction of Munc 18 with syntaxin1 in ASMko versus wt samples.
(1.08 MB EPS)

Figure S3 Accumulation of uncatabolized lipid substrates in hippocampal neurons of ASMko mice. A, B, C, D: light-microscopic micrographs of semithin sections through the CA1 region of wt (A,C) and ASMko (B,D) mice depicting the cell bodies of hippocampal pyramidal neurons at 1 month (A,B) and 7 months (C,D) of age. E: Inset of the area depicted with a rectangle in B where the lipid inclusions are shown at a higher magnification. Nu, nucleus. Scale bar: 20 µm.
(5.84 MB EPS)

Figure S4 Syntaxin1A Purification. Protein profiles were visualized using Coomassie blue staining of the different fractions of the purification protocol. Molecular weight markers used in the SDS-PAGE are indicated on the left in kDa.
(0.51 MB EPS)

Figure S5 Effects of Se Treatment on the Lipid Composition of Wt Synaptosomes. Levels of cholesterol (Chol), phospholipids (PE, PC and PS), Ceramide (Cer), SM and Se in wt synaptosomes treated or not with 100 µM Se for 2.5 hours at 370 C, followed by dilution and recentrifugation before lipid analysis (mean±SD; n = 3).
(0.76 MB EPS)
